# Markers of breast cancer stromal fibroblasts in the primary tumour site associated with lymph node metastasis: a systematic review including our case series

**DOI:** 10.1042/BSR20130060

**Published:** 2013-12-12

**Authors:** Maria Aparecida Azevedo Koike Folgueira, Simone Maistro, Maria Lucia Hirata Katayama, Rosimeire Aparecida Roela, Fiorita Gonzales Lopes Mundim, Suely Nanogaki, Geertruida H. de Bock, M. Mitzi Brentani

**Affiliations:** *Departamento de Radiologia e Oncologia, Disciplina de Oncologia, Faculdade de Medicina da Universidade de São Paulo (LIM-24), São Paulo, Brasil; †Department of Epidemiology, University Medical Center Groningen, University of Groningen, Groningen, The Netherlands.; ‡Departamento de Anatomia Patológica, Hospital A.C. Camargo, São Paulo, Brasil; §Departamento de Patologia, Universidade do Vale do Sapucaí, Pouso Alegre, MG, Brasil

**Keywords:** breast cancer, cancer-associated fibroblasts, caveolin 1, lymph node metastasis, MMP13, podoplanin, CAFs, cancer-associated fibroblasts, CAV1, caveolin-1, ECM, extracellular matrix, HG3, histological grade 3, LGALS1, lectin, galactoside-binding, soluble, 1, MMP13, matrix metalloproteinase 13, OR, odds ratio, PDPN, podoplanin, PLAU, plasminogen activator, urokinase, PLAUR, plasminogen activator, urokinase receptor, SI, staining index, THBS1, thrombospondin 1, TIMP2, tissue inhibitor of metalloproteinases-2, TMA, tissue microarray assembly, uPA, urokinase-type plasminogen activator

## Abstract

CAFs (cancer-associated fibroblasts), the most abundant cell type in breast cancer stroma, produce a plethora of chemokines, growth factors and ECM (extracellular matrix) proteins, that may contribute to dissemination and metastasis. Axillary nodes are the first metastatic site in breast cancer; however, to the present date, there is no consensus of which specific proteins, synthesized by CAFs, might be related with lymph node involvement. The purpose of this study was to perform a systematic review of CAF biomarkers associated with the presence of regional metastasis. PubMed was searched using the words: ‘breast cancer’ and ‘lymph node’ and fibroblast or stroma or microenvironment. After exclusions, eight studies evaluating biomarkers immunoexpression in CAFs and lymph node status were selected. Biomarkers evaluated in these studies may be divided in two groups, according to their ontology: extracellular matrix components [MMP13 (matrix metalloproteinase 13), TIMP2 (tissue inhibitor of metalloproteinases-2), THBS1 (thrombospondin 1), LGALS1 (lectin, galactoside-binding, soluble, 1)] and response to wounding [PDPN (podoplanin), PLAU (plasminogen activator, urokinase), PLAUR (plasminogen activator, urokinase receptor), CAV1 (caveolin 1), THBS1, LGALS1]. A positive expression of MMP13 and LGALS1 in CAFs was associated with enhanced OR (odds ratio) for regional metastasis. Contrariwise, CAV1 positive staining of fibroblasts was associated with decreased OR for nodal involvement. Expression of MMP13, PDPN and CAV1 was further tested in a new series of 65 samples of invasive ductal breast carcinomas by immunohistochemistry and no association between biomarkers expression in CAFs and nodal status was found. It was suggested that breast cancer subtypes may differentially affect CAFs behaviour. It would be interesting to evaluate the prognostic significance of these biomarkers in CAFs from different tumour types.

## INTRODUCTION

There is increasing evidence that breast cancer behaviour reflects an interconnection between the malignant epithelial compartment and the surrounding microenvironment.

In contrast with normal physiological stroma, which maintains epithelial cell polarity and inhibits epithelial cell growth and transformation, tumour stroma may stimulate the whole tumour development process [1]. In fact, presence of stromal desmoplastic reaction and stroma-rich tumours were both significantly associated with worse prognosis in breast cancer [2, 3]. In addition, a recent study classifying tumour stroma found that collagen and fibroblast dominant groups were associated with poor outcome [4].

Fibroblasts [also called CAFs (cancer-associated fibroblasts)], the most abundant cell type in breast cancer stroma, produce a plethora of chemokines, growth factors and ECM (extracellular matrix) proteins, all of which may contribute to increased dissemination and metastasis [5].

Although no major morphologic differences are recognized between CAFs and their normal counterparts, i.e. fibroblasts from the matched adjacent uninvolved mammary tissue or reduction mammoplasties, microarray and proteomic analysis revealed distinct mRNAs and protein expression profiles between them [6–11].

In co-culture assays it was shown that CAFs may induce epithelial cell proliferation and migration [12–15] and in animal models it was reported that activated fibroblasts may act as cofactors in cell transformation, promoting tumour development from probably initiated, but still apparently normal mammary cells [16]. In addition, a dynamic bidirectional signalling between CAFs and breast cancer cells was detected, indicating that the former may influence the transcriptional profile of breast cancer cells and vice versa, the latter may affect the gene signature of CAFs [14, 17].

It was reported that gene expression patterns from tumour stromal cells may be correlated with disease-free survival in breast cancer [7]. However, only a few works have directly examined stromal cells signature through microdissected samples [7, 18], whereas others have used different models as fibroblasts primary culture [10], tumour samples with differential contents of stroma tissue [19] or a panel of previously identified ECM-related genes [20], to estimate the influential effect of stroma in breast cancer behaviour.

Axillary nodes status is one of the established prognostic factors in breast cancer. However, to the present date, there is no consensus of which specific proteins, synthesized by CAFs, might be related with lymph node involvement. To address this issue a systematic review of studies evaluating CAF biomarkers associated with the presence of regional metastasis was undertaken and selected markers were further analysed in samples from a group of patients with breast cancer with involved or uninvolved nodes by immunohistochemistry.

## MATERIALS AND METHODS

### Search strategy

PubMed was searched by two reviewers (SM and MLHK or RAR) for relevant trials using the following words: ‘breast cancer’ and ‘lymph node’ and fibroblast or stroma or microenvironment. Electronic search was performed on March 4, 2013.

### Inclusion criteria

**Figure 1:**
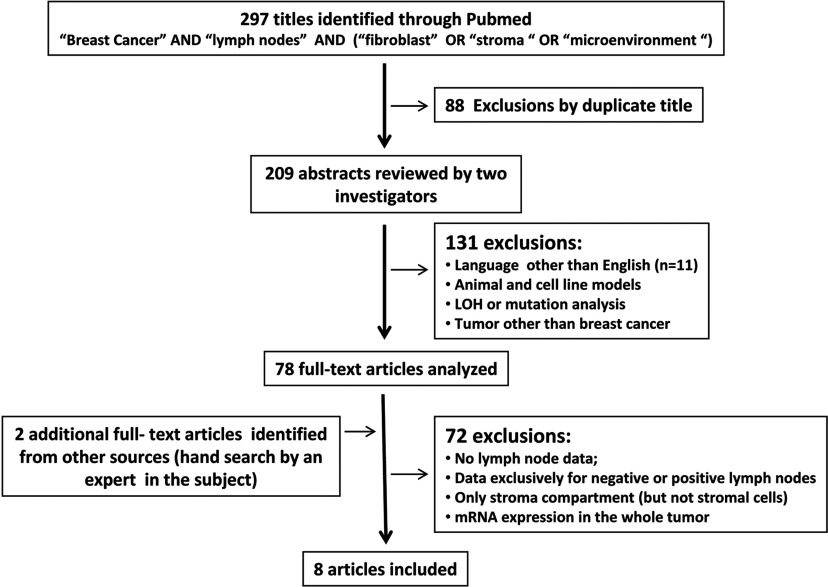
Stages of the search strategy

Studies presenting data on specific biomarker in CAFs or stromal cells in the primary tumour and involvement of lymph nodes were included. Studies evaluating the stroma compartment without a specific reference to each cellular element were excluded, as well as studies employing mRNA analysis by RT–PCR or microarray without microdissection. At first, we did not exclude studies evaluating nodal status and stromal cells gene expression profiles, however, as tumour samples were not microdissected and results were based on indirect analysis, these works were then excluded. Studies evaluating fibroblasts for loss of heterozygosis and point mutations, studies in cell culture or animal models or other diseases than breast cancer and studies without reference to the nodal status of patients were also excluded. Only studies in English language with an available abstract through Pubmed were included (figure 1).

Seven reviews were identified, three of which, written before 1997, were not available as a full-text paper [21–23]. The other four reviewed pathways in breast cancer development: RANK–RANKL system, CCN6 and TGFβ (transforming growth factor β) or mainly dealt with immune system cells and were not further analysed [24–27]. All the cited references were checked. No systematic reviews were identified.

### Patients

Breast cancer samples from 65 patients undergoing breast surgery at Hospital Samuel Libânio, in Pouso Alegre, MG, Brasil from 1997 to 2005, were retrospectively analysed. This study was approved by the Institutional Ethics Committee of Hospital Samuel Libânio (no. 1119/09). Mean age of patients was 57.78±13.9 years (range: 23–84 years). All patients were diagnosed with IDCs (invasive ductal carcinomas), 54% of whom, presenting involved axillary nodes, and 24.6, 40.0 and 32.9% with disease clinical stages I, II or III, respectively (two unknown). ER (oestrogen receptor), PR (progesterone receptor) and HG3 (histological grade 3) were detected in 58.7, 50 and 40% of the samples, respectively.

### TMA (tissue microarray assembly)

Paraffin blocks of the primary tumours were reviewed for the selection of a representative area of tumour stroma, which was then collected using a Manual Tissue Arrayer-1 (Beecher Instruments). Samples were orderly arranged in a grid and the first core was represented by a fragment of normal liver used as a reference. Samples of 3 μm were then placed on slides using special adhesives (Instrumedics Inc.). To obtain two levels of the same samples on the TMA, two sets of tumour triplets were assembled on the slides with a gap of 40 cuts between them. A second TMA was assembled containing mainly cancer cells.

### Immunohistochemistry

Immunohistochemistry reactions were analysed using optical microscope Nikon Eclipse E200 ((Nikon Instruments), magnification: 400×, aperture of the objective lenses: 40×/0.65 (∞/0.17- WD0.65) and temperature: 20°C/68 °F equipped with Motic 1000 camera). The images were acquired by Motic Images Plus 2.0. Tissue sections were deparaffinized in xylene and rehydrated with graded ethanol. Antigen retrieval was performed using a pressure cooker in citrate buffer pH 6.0, followed by endogenous peroxidase blocking with hydrogen peroxidase solution (3%). Sections were then incubated with primary monoclonal antibodies: MMP13 (matrix metalloproteinase 13) (1:150, diluted Clone VIIIA2 from Lab Vision Corporation), CAV1 (caveolin-1; 1:800, from Cell Signaling Technology) and PDPN (podoplanin; clone D2–40, from Signet Laboratories). After primary antibody incubation and pos primary blocking a polymer-peroxidase (Novolink, Novocastra, Leika Biosystems) amplification step was done. Antigen detection was carried out in a solution containing 3,3-diaminobenzidine (Sigma) and 6% (v/v) H_2_O_2_. Counterstaining was performed with Harris haematoxylin (Merck).

A three-grade system was used to evaluate PDPN and CAV1 expression in stromal cells. CAV1 staining in stromal cells was graded as follows: negative expression (−), ≤ 5%; low expression (+), 5–50%; and high expression (++), >50%. PDPN was evaluated as: negative expression (−), ≤ 10%; low expression (+), 10–50%; and high expression (++), >50% [28,29]. Both the intensity and percentage of positivity were assessed to define the expression of MMP13. No brown particle staining: 0; light brown particle in cytoplasm: 1; moderate brown particle: 2; and dark brown particle: 3. The percentage of positive cells was classified into four groups. 1: <25%; 2: 25–50%; 3, 51–75% and 4,>75% positive cells. The SI (staining index), the sum of the intensity and the percentage of positive staining, was used to define high (SI ≥ 6) or low (SI ≤ 6) expression of MMP13 [30].

### Statistical analysis

Pearson *χ*^2^ and OR (odds ratio) were calculated using SPSS 11.0 version software (SPSS), and *P* value<0.05 was considered significant.

## RESULTS

The aim of this study was to address the following question: Does the expression of stromal cell biomarkers increase the risk of presenting lymph node metastasis in breast cancer?

To accomplish this aim a comprehensive literature search for all studies addressing the relationship between biomarker expression in stromal cells and lymph node involvement was conducted (Figure 1).

At first, 297 titles were retrieved from which, six fulfilling the inclusion criteria. Two manuscripts [31,32] were subsequently added by MMB after reviewing her archives, one of them, a reference in one of the 78 full texts analysed [33]. Hence, eight manuscripts were reviewed. Most exclusions were taken for absence of lymph node data, exclusive analysis in node positive or negative disease; analysis of the stroma compartment but not specifically of the stromal cells. In total, 1408 patients were analysed and their clinical characteristics appear in Table 1.

**Table 1:** Clinical and tumour characteristics IDC, invasive ductal carcinoma; HG3, histological grade 3; N^+^, positive node.

Author	Biomarkers	City, Country	Sample size (*n*)	Age (years)	IDC (%)	HG3 (%)	N^+^ (%)	ER^+^ (%)	PR^+^ (%)
Zhang et al. [30]	MMP13	Tianjin, China	*n*=263	Median: 50.3	100	29	51	62	62
Nakopoulou et al.[36]	TIMP2	Athens, Greece	*n*=136	Mean: 57.09 (25–87)	79	25	58	54	50
Ioachim et al.[35]	THBS1	Ioannina, Greece	*n*=124	-	61	39	66	68	60
Jung et al. [34]	LGALS1	Jinju, Korea	*n*=105	Mean: 39.3±9.8	41	-	40	87	87
Schoppmann et al. [32]	PDPN	Vienna, Austrian	*n*=367	Mean: 61±13	87	40	43	79	-
Pula et al.[28]	PDPN	Wroclaw, Poland	*n*=117	Mean: 56.6±11.3 (30–83)	100	35	49	76	65
Dublin et al.[31]	PLAU, PLAUR, PAI1	U.K.	*n*=142	Median: 56 (28–86)	62	34	63	-	-
Witkiewicz et al. [37]	CAV1	Michigan, U.S.A.	*n*=154	Median: 59.5 (28–96)	-	46	52	70	56
Present Series	MMP13, PDPN, CAV1	Pouso Alegre, Brazil	*n*=65	Mean: 57.7±13.9 (23–84)	100	40	54	59	50

Biomarkers evaluated in the reviewed studies may be divided into two groups, according to their ontology: extracellular matrix components [MMP13, TIMP2 (metallopeptidase inhibitor 2), THBS1 (thrombospondin 1), LGALS1 (lectin, galactoside-binding, soluble, 1)] and response to wounding [PDPN, PLAU (plasminogen activator, urokinase), PLAUR (plasminogen activator, urokinase receptor), CAV1, LGALS1 and THBS1].

In the first group, positive expression of MMP13 and LGALS1 in CAFs was associated with enhanced OR for regional metastasis [30, 34]. Contrariwise, positive expression of THBS1 showed a trend towards decreased OR for nodal involvement and TIMP2 expression was not associated with lymph node metastasis [35,36]. A combined evaluation of the expression of these extracellular matrix components (MMP13, TIMP2, LGALS1 and THBS1) in CAFs was now performed and found to be associated with an increased OR for involved nodes (table 2).

**Table 2:** Immunoexpression of biomarkers in breast cancer stromal cells and OR for positive nodes (N^+^) (**1**) staining index (SI: product of intensity and percentage of positive staining, high:SI ≥ 6, at least 25% cells with moderate staining); (**2**) Positive cells >11%; (**3**) Combined scores of the sum of staining intensity and percentage positive cells: (−)=no staining, (+)=weak, (++)=moderate, (+++)=strong (**4**) strong: at least 30% positive cells; (**5**) expression ≥10% of the tumour stroma; (**6**) positive: ratio of podoplanin-positive stromal area to overall stromal area ≥10%; (**7**) negative (no staining), weak (definite but weak staining), strong (intense staining); (**8**) absent (no staining), present (either diffuse weak to strong staining); (**9**) expression ≥50% of the tumour stroma. Combined OR for N^+^ considering studies reporting analysis of more than one biomarker: **ECM** (MMP13/TIMP2/LGALS1/THBS1); **PDPN** (b+c) and **Response to wounding** (LGALS1/THBS1/PDPN(b+c)/CAV1/PLAU or PLAUR).

(a)						
Study	Biomarker		N^+^ (%)	N^−^ (%)	OR (95%)	*P*
Zhang et al. [30]	MMP13(a)	High^(1)^	66	43	2.57 (1.56–4.23)	< 0.001
Nakopoulou et al. [36]	TIMP2	>11%^(2)^	73	82	0.58 (0.24–1.34)	0.201
Ioachim et al. [35]	THBS1	(+/++/+++)^(3)^	41	62	0.44 (0.19-1.03)	0.06
Jung et al. [34]	LGALS1	Strong^(4)^	70	51	2.31 (1.02–5.23)	0.04
***ECM (MMP13/TIMP2/LGALS1/THBS1)***		Positive	63	55	1.41 (1.02–1.96)	0.04
Schoppmann et al. [32]	PDPN(b)	≥10%^(5)^	4	12	0.32 (0.14–0.77)	0.008
Pula et al. [28]	PDPN(c)	≥10%^(6)^	91	73	3.87 (1.31-1.42)	0.010
	*PDPN (b + c)*	≥10%	27	26	1.09 (0.72–1.63)	0.67
Dublin et al. [31]	PLAU	Strong^(7)^	46	41	1.23 (0.58–2.61)	0.58
Dublin et al. [31]	PLAUR	Strong^(7)^	54	66	0.61 (0.28–1.31)	0.20
Dublin et al. [31]	PAI1	Strong^(7)^	17	11	1.61 (0.53–4.86)	0.39
Witkiewicz et al. [37]	CAV1(d)	Present^(8)^	46	76	0.27 (0.12–0.63)	0.002
***Response to wounding (LGALS1/THBS1/PDPN(b,c)/CAV1/PLAU)***		Positive	39	40	0.97 (0.75–1.27)	0.83
***Response to wounding (LGALS1/THBS1/PDPN/(b,c)/CAV1/PLAUR)***		Positive	40	42	0.93 (0.71–1.21)	0.60
(b)						
Study	Biomarker		N^+^	N^−^	OR (95%)	*P*
Present results	MMP13(e)	High^(1)^	63	64	0.97 (0.34–2.78)	0.958
	PDPN(f)	≥10%^(6)^	79	65	2.03 (0.65 – 6.29)	0.361
	CAV1(g)	>50%^(9)^	41	45	0.84 (0.30–2.33)	0.74
**Metanalysis (previous studies + present results)**
	***MMP13 (a+e) n=324***	High	66	47	2.15 (1.38-3.37)	0.001
	***PDPN (b+c+f) n=546***	≥10%	34	30	1.25 (0.87–1.79)	0.128
	***CAV1 (d+g) n=165***	>50%	44	64	0.43 (0.23–0.81)	0.007

In the group of biomarkers involved in response to wounding (PDPN, PLAU, PLAUR, CAV1, THBS1 and LGALS1), two studies evaluated PDPN expression in CAFs, with contrasting results: one of them showed an increased and the other one a decreased OR for lymph node involvement [28, 32]. In addition, one study evaluated the expression of three proteins, PLAU, PLAUR and PAI1 (plasminogen activator inhibitor-1), in CAFs from the same tumour samples, but found no association with lymph node metastasis [31]. In another work, CAV1 positive staining of fibroblasts was associated with decreased OR for axillary involvement [37]. A combined evaluation of expression of response to wounding biomarkers was not associated with differential OR for involved nodes, as shown in Table 2.

**Figure 2:**
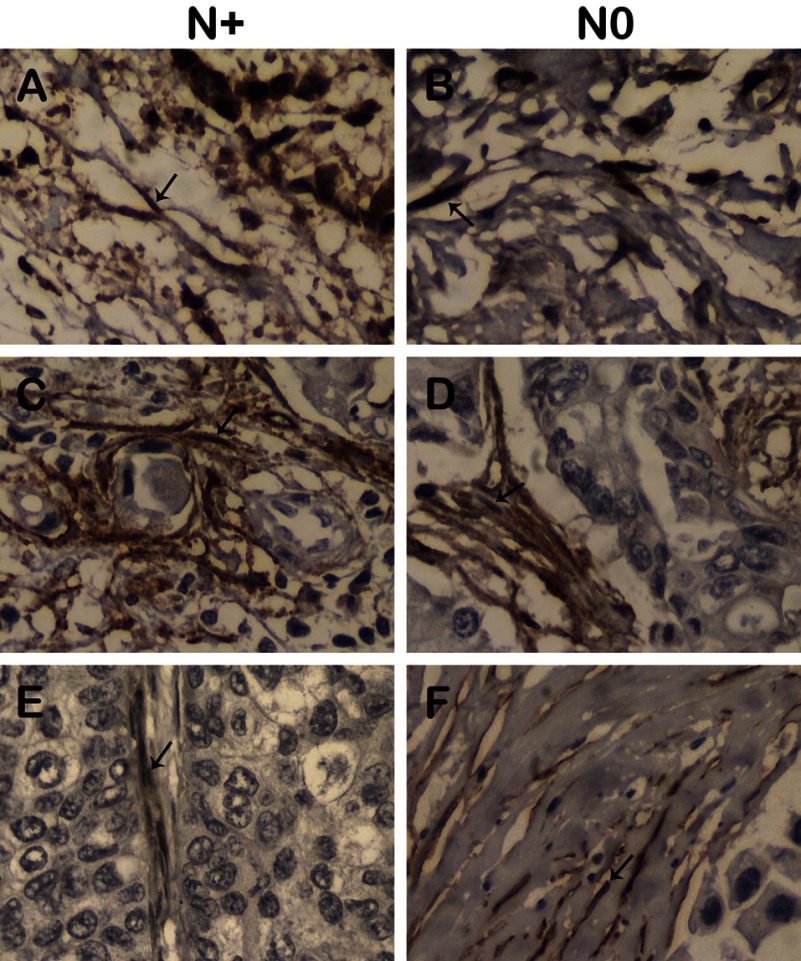
Immunohistochemical staining of MMP13 (A and B), PDPN (C and D) and CAV1 (E and F) in CAFs from breast cancer samples from patients with involved (N^+^) and uninvolved (N0) nodes Positive immunostaining indicated (arrows). Original magnification: MMP13: ×100, CAV1: ×400, PDPN: ×100.

After the systematic review, three biomarkers found associated with nodal metastasis, MMP13, PDPN and CAV1, were selected for further evaluation in a group of 65 patients with involved or uninvolved nodes (56 and 44% of them, respectively). Expression analysis was determined in stroma-rich areas by immunohistochemistry reactions in TMA, as shown in Figure 2.

Metalloproteinase 13 expression was detected as a cytoplasmic staining in both CAFs, as well as malignant cells, in all tumour samples, except one. In CAFs, MMP13 strong expression evaluated as staining index ≥ 6 was observed in 63.9% of the samples (39/61). PDPN expression was also detected as a cytoplasmic staining in 73% of CAFs (46/63). It was also observed in malignant cells in two tumour samples. Caveolin expression was detected on the membrane and/or cytoplasm of CAFs, exclusively (but not in malignant cells). In addition, caveolin expression was detected in at least 50% of CAFs in 42.6% of the samples (26/61).

In the present work no association between expression of these three proteins (MMP13, PDPN and CAV1) and lymph node involvement was found (table 2).

A metanalysis including present and previous results indicate that PDPN expression in CAFs is not associated with node involvement while MMP13 and CAV1 high expression are associated with increased and decreased OR, respectively, for axillary involvement (table 2).

## DISCUSSION

Some studies have pointed out that a reactive stroma may negatively affect disease outcome in breast cancer [7, 20]. However, tumour microenvironment reflects stromal cells behavior and extracellular matrix content, which is determined by both stromal and cancer cells. Our aim was to evaluate which biomarkers expressed by stromal cells might be associated with the risk of positive nodes in breast cancer.

Two studies have previously directly analysed microdissected stromal cells from breast cancer samples describing stromal signatures predictive of poor outcome, but none of them specifically reported nodal involvement [7, 18]. One of these works described a reactive stroma profile, characterized by an increased expression of genes encoding ECM components, proteolytic enzymes and adhesion receptors [18]. In addition, some other stromal signatures indirectly identified, i.e., not from microdissected stromal cells, were also shown to have prognostic value [19, 20, 38].

As tumour metastasis involves a series of steps, including a reactive stroma, partly secreted by stromal cells, a relative high number of manuscripts were expected in this systematic review. However, after exclusions, eight studies were retrieved, all performed in tissue samples examined by immunohistochemistry. In these works, protein expression was specifically described in CAFs or stromal cells.

The studies identified in the present review, mainly focused on classical markers synthesized by stromal cells, as extracellular matrix components (MMP13, TIMP2, THBS1 and LGALS1) and proteins involved in response to wounding (PDPN, PLAUR, PLAU, CAV1, LGALS1 and THBS1). However, except for PDPN determinations, we could identify only one work analysing expression of each of these markers.

Extracellular matrix signatures were previously shown to be related with tumour outcome and a variety of metagenes were described, including metalloproteases, whose expression levels were associated with breast cancer survival [18, 20, 38]. In the works reviewed in this analysis [30, 34-36], a positive expression of MMP13 and LGALS1 by stromal cells was associated with increased OR for nodal involvement, in contrast with positive THBS1 expression that showed a trend towards association with decreased OR for node positivity. A combined analysis of ECM components was performed, however, statistical significance to detect nodal involvement was not improved, probably because it involves expression of proteins with different functions and odds ratios in opposite directions.

Matrix metalloproteases are proteins involved in the breakdown of extracellular matrix. Among them, a higher immunoexpression of MMP13 (collagenase 3) in stromal cells was associated with positive nodes in breast cancer [30]. In agreement, MMP13 expression by intratumoural fibroblasts was significantly associated with shorter relapse-free survival in luminal A tumours [39]. However, MMP13 was also detected in the cytoplasm of cancer cells [40] and its high immunoexpression was also associated with poor outcome [30]. In addition, whole tumour MMP13 protein amount, analysed from tumour lysates and reflecting tumour and stromal cells, as well as ECM content, was not correlated with nodal status [41]. In accordance, MMP13 high expression in CAFs was not associated with axillary involvement in the present work. Hence, there is no consensus and it seems likely that a fine tune in MMP13 tumour content is involved in regulating nodal metastasis.

TIMPs are endogenous regulators of MMPs with contradictory activities as inhibiting MMPs and promoting tumour cell growth. In breast cancer, TIMP2 immunoexpression may be localized in cancer as well as stromal cells, however mRNA *in situ* hybridization indicates a lower TIMP2 synthesis in stromal cells than in tumour cells [42]. It was shown that TIMP2 protein expression in stroma cells may be inversely correlated with tumour size; however, there was no association with lymph node involvement [36].

Galectin 1 is a beta galactoside-binding protein which may be involved in tumour progression. Galectin 1 is synthesized by cancer as well as stromal cells and its overexpression in both cell types in breast cancer samples, indistinctly, was also associated with high tumour grade, a poor prognosis marker [43]. In agreement, Galectin 1 stromal cell expression was another predictive marker of node involvement [34].

Thrombospondin (THBS1) is an antiangiogenic extracellular matrix protein that inhibits tumour growth and metastasis in animals. Although there is no consensus of its clinical relevance, there is a trend towards an inverse correlation between increased THBS1 expression in tumour stroma (evaluated as stromal area) or stromal cells and nodal positivity [35, 44]. These studies were not analysed together in the present systematic review as one of them [44] evaluated THBS1 in the stromal area but not specifically in the stroma fibroblasts.

PDPN is a type-I integral membrane glycoprotein, which expression was shown up-regulated in a number of different cancers [45]. Two studies evaluating stromal cells PDPN immunoexpression, using equivalent cut-offs, reported contrasting results. PDPN loss of positivity in stromal cells was associated with presence of lymph node metastasis in one [32] but not in another study [28]. In the present work, PDPN expression in CAFs was not related with axillary involvement, further indicating that its relationship remains elusive.

The uPA (urokinase-type plasminogen activator/PLAU) and its main inhibitor PAI1 play key roles in tumour-associated processes and their elevated expression is correlated with negative outcomes in node negative breast cancer. However, clinical utility of these molecules as prognostic markers is limited by the use of ELISA tests for their detection in fresh or frozen tissue [46]. It is unclear whether their relative levels in the tumour stroma or in the tumour cells themselves are the most relevant to patient outcome. Using microdissected stromal and cancer cells it was shown that similar expression levels occur in both compartments and that quantity of tumour stroma in the tumour tissue specimen is not relevant [47]. However, in node negative patients, immunohistochemistry study has shown that both fibroblastic PAI1 and diffuse stromal uPA are associated with local recurrence [48] and even though fibroblastic expression of uPA and uPAR (urokinase-type plasminogen activator receptor) and strong PAI1 expression was related to the presence of invasion or tumour size, it was not associated with node metastasis [31].

CAV1 is an important component of caveolae membranes and loss of CAV1 in the fibroblast compartment, rather than within breast cancer tumour cells was shown to be an independent predictor of lymph node metastasis and poor outcome [37]. In addition, it was reported that high levels of CAV1 in the stromal breast cancer tissue were associated with reduced frequency of nodal metastasis and improved survival [49]. These results, however, were not confirmed in the present series of IDC nor by other authors [29] who could neither verify an association between loss of stromal CAV1 and the occurrence of positive lymph nodes. Discrepancies may in part be due to tumour heterogeneity. Breast cancer is believed to be a heterogeneous disease and expression of stromal biomarkers may be connected to particular subtypes of breast carcinoma, as recently suggested [50]. Variations in expression of biomarkers in fibroblasts between different histological types was reported, demonstrating that invasive ductal breast carcinomas have high global expressions of MMPs, including MMP13, and TIMPs compared with the remaining types of breast carcinoma [51]. Furthermore, CAFs are identified by a lot of markers such as αSMA, S100A4, possibly because they originated from heterogeneous population [52], raising the possibility that specific subtypes of CAFs may be associated with different frequencies of the chosen markers.

In conclusion, only few works specifically evaluated biomarker expression in CAFs and their relation with nodal involvement. These studies were concerned in evaluating expression of extracellular matrix components and proteins involved in response to wounding, which are believed to be mainly synthesized and secreted by fibroblasts. In summary, there is some evidence that a high expression of MMP13 and PDPN may be related with increased OR for positive nodes and a positive expression of CAV1 with decreased OR for positive nodes. Breast cancer subtypes based on tumour transcriptional profile were shown to have prognostic importance. Hence, it would be interesting to analyse CAFs role in tumour aggressiveness in each specific breast carcinoma subtype, as well the relationship between stromal biomarkers and different CAF subsets.

## References

[1] Bhowmick N. A., Moses H. L. (2005). Tumor–stroma interactions. Curr. Opin. Genet. Dev..

[2] Hasebe T., Sasaki S., Imoto S., Ochiai A. (2000). Proliferative activity of intratumoral fibroblasts is closely correlated with lymph node and distant organ metastases of invasive ductal carcinoma of the breast. Am. J. Pathol..

[3] de Kruijf E. M., van Nes J. G., van de Velde C. J., Putter H., Smit V. T., Liefers G. J., Kuppen P. J., Tollenaar R. A., Mesker W. E. (2011). Tumor–stroma ratio in the primary tumor is a prognostic factor in early breast cancer patients, especially in triple-negative carcinoma patients. Breast Cancer Res. Treat..

[4] Ahn S., Cho J., Sung J., Lee J. E., Nam S. J., Kim K. M., Cho E. Y. (2012). The prognostic significance of tumor-associated stroma in invasive breast carcinoma. Tumour Biol..

[5] Allen M., Louise Jones J. (2011). Jekyll and Hyde: the role of the microenvironment on the progression of cancer. J. Pathol..

[6] Allinen M., Beroukhim R., Cai L., Brennan C., Lahti-Domenici J., Huang H., Porter D., Hu M., Chin L., Richardson A. (2004). Molecular characterization of the tumor microenvironment in breast cancer. Cancer Cell..

[7] Finak G., Bertos N., Pepin F., Sadekova S., Souleimanova M., Zhao H., Chen H., Omeroglu G., Meterissian S., Omeroglu A. (2008). Stromal gene expression predicts clinical outcome in breast cancer. Nat. Med..

[8] Singer C. F., Gschwantler-Kaulich D., Fink-Retter A., Haas C., Hudelist G., Czerwenka K., Kubista E. (2008). Differential gene expression profile in breast cancer-derived stromal fibroblasts. Breast Cancer Res. Treat..

[9] Bauer M., Su G., Casper C., He R., Rehrauer W., Friedl A. (2010). Heterogeneity of gene expression in stromal fibroblasts of human breast carcinomas and normal breast. Oncogene.

[10] Tchou J., Kossenkov A. V., Chang L., Satija C., Herlyn M., Showe L. C., Puré E. (2012). Human breast cancer associated fibroblasts exhibit subtype specific gene expression profiles. BMC Med. Genomics.

[11] Campos L. T., Brentani H., Roela R. A., Katayama M. L., Lima L., Rolim C. F., Milani C., Folgueira M. A., Brentani M. M. (2013). Differences in transcriptional effects of 1α,25 dihydroxyvitamin D3 on fibroblasts associated to breast carcinomas and from paired normal breast tissues. J. Steroid Biochem. Mol. Biol..

[12] Hu M., Yao J., Carroll D. K., Weremowicz S., Chen H., Carrasco D., Richardson A., Violette S., Nikolskaya T., Nikolsky Y. (2008). Regulation of *in situ* to invasive breast carcinoma transition. Cancer Cell.

[13] Krtolica A., Parrinello S., Lockett S., Desprez P. Y., Campisi J. (2001). Senescent fibroblasts promote epithelial cell growth and tumorigenesis: a link between cancer and aging. Proc. Natl. Acad. Sci. U.S.A..

[14] Santos R. P., Benvenuti T. T., Honda S. T., Del Valle P. R., Katayama M. L., Brentani H. P., Carraro D. M., Rozenchan P. B., Brentani M. M., de Lyra E. C. (2011). Influence of the interaction between nodal fibroblast and breast cancer cells on gene expression. Tumour Biol..

[15] Casbas-Hernandez P., Fleming J. M., Troester M. A. (2011). Gene expression analysis of *in vitro* cocultures to study interactions between breast epithelium and stroma. J. Biomed. Biotechnol..

[16] Kuperwasser C., Chavarria T., Wu M., Magrane G., Gray J. W., Carey L., Richardson A., Weinberg R. A. (2004). Reconstruction of functionally normal and malignant human breast tissues in mice. Proc. Natl. Acad. Sci. U.S.A..

[17] Rozenchan P. B., Carraro D. M., Brentani H., de Carvalho Mota L. D., Bastos E. P., e Ferreira E. N., Torres C. H., Katayama M. L., Roela R. A., Lyra E. C. (2009). Reciprocal changes in gene expression profiles of cocultured breast epithelial cells and primary fibroblasts. Int. J. Cancer.

[18] Planche A., Bacac M., Provero P., Fusco C., Delorenzi M., Stehle J. C., Stamenkovic I. (2011). Identification of prognostic molecular features in the reactive stroma of human breast and prostate cancer. PLoS ONE.

[19] Bianchini G., Qi Y., Alvarez R. H., Iwamoto T., Coutant C., Ibrahim N. K., Valero V., Cristofanilli M., Green M. C., Radvanyi L. (2010). Molecular anatomy of breast cancer stroma and its prognostic value in estrogen receptor-positive and -negative cancers. J. Clin. Oncol..

[20] Bergamaschi A., Tagliabue E., Sørlie T., Naume B., Triulzi T., Orlandi R., Russnes H. G., Nesland J. M., Tammi R., Auvinen P. (2008). Extracellular matrix signature identifies breast cancer subgroups with different clinical outcome. J. Pathol..

[21] Rochefort H. (1990). Biological and clinical significance of cathepsin D in breast cancer. Semin. Cancer Biol..

[22] Harris A. L., Fox S., Bicknell R., Leek R., Relf M., LeJeune S., Kaklamanis L. (1994). Gene therapy through signal transduction pathways and angiogenic growth factors as therapeutic targets in breast cancer. Cancer.

[23] Price J. E. (1996). Metastasis from human breast cancer cell lines. Breast Cancer Res. Treat..

[24] Hanada R., Hanada T., Sigl V., Schramek D., Penninger J. M. (2011). RANKL/RANK-beyond bones. J. Mol. Med (Berl.)..

[25] Huang W., Pal A., Kleer C. G. (2011). On how CCN6 suppresses breast cancer growth and invasion. J. Cell Commun. Signal..

[26] Bierie B., Moses H. L. (2009). Gain or loss of TGFbeta signaling in mammary carcinoma cells can promote metastasis. Cell Cycle.

[27] de Sousa M. (2011). An outsider's perspective–ecotaxis revisited: an integrative review of cancer environment, iron and immune system cells. Integr. Biol..

[28] Pula B., Jethon A., Piotrowska A., Gomulkiewicz A., Owczarek T., Calik J., Wojnar A., Witkiewicz W., Rys J., Ugorski M. (2011). Podoplanin expression by cancer-associated fibroblasts predicts poor outcome in invasive ductal breast carcinoma. Histopathology.

[29] Qian N., Ueno T., Kawaguchi-Sakita N., Kawashima M., Yoshida N., Mikami Y., Wakasa T., Shintaku M., Tsuyuki S., Inamoto T., Toi M. (2011). Prognostic significance of tumor/stromal caveolin-1 expression in breast cancer patients. Cancer Sci..

[30] Zhang B., Cao X., Liu Y., Cao W., Zhang F., Zhang S., Li H., Ning L., Fu L., Niu Y. (2008). Tumor-derived matrix metalloproteinase-13 (MMP-13) correlates with poor prognoses of invasive breast cancer. BMC Cancer.

[31] Dublin E., Hanby A., Patel N. K., Liebman R., Barnes D. (2000). Immunohistochemical expression of uPA, uPAR, and PAI-1 in breast carcinoma. Fibroblastic expression has strong associations with tumor pathology. Am. J. Pathol..

[32] Schoppmann S. F., Berghoff A., Dinhof C., Jakesz R., Gnant M., Dubsky P., Jesch B., Heinzl H., Birner P. (2012). Podoplanin-expressing cancer-associated fibroblasts are associated with poor prognosis in invasive breast cancer. Breast Cancer Res. Treat..

[33] Hemsen A., Riethdorf L., Brünner N., Berger J., Ebel S., Thomssen C., Jänicke F., Pantel K. (2003). Comparative evaluation of urokinase-type plasminogen activator receptor expression in primary breast carcinomas and on metastatic tumor cells. Int. J. Cancer.

[34] Jung E. J., Moon H. G., Cho B. I., Jeong C. Y., Joo Y. T., Lee Y. J., Hong S. C., Choi S. K., Ha W. S., Kim J. W. (2007). Galectin-1 expression in cancer-associated stromal cells correlates tumor invasiveness and tumor progression in breast cancer. Int. J. Cancer.

[35] Ioachim E., Damala K., Tsanou E., Briasoulis E., Papadiotis E., Mitselou A., Charhanti A., Doukas M., Lampri L., Arvanitis D. L. (2012). Thrombospondin-1 expression in breast cancer: prognostic significance and association with p53 alterations, tumour angiogenesis and extracellular matrix components. Histol. Histopathol..

[36] Nakopoulou L., Katsarou S., Giannopoulou I., Alexandrou P., Tsirmpa I., Panayotopoulou E., Mavrommatis J., Keramopoulos A. (2002). Correlation of tissue inhibitor of metalloproteinase-2 with proliferative activity and patients' survival in breast cancer. Mod. Pathol..

[37] Witkiewicz A. K., Dasgupta A., Sotgia F., Mercier I., Pestell R. G., Sabel M., Kleer C. G., Brody J. R., Lisanti M. P. (2009). An absence of stromal caveolin-1 expression predicts early tumor recurrence and poor clinical outcome in human breast cancers. Am. J. Pathol..

[38] West R. B., Nuyten D. S., Subramanian S., Nielsen T. O., Corless C. L., Rubin B. P., Montgomery K., Zhu S., Patel R., Hernandez-Boussard T. (2005). Determination of stromal signatures in breast carcinoma. PLoS Biol..

[39] González L. O., Corte M. D., Junquera S., González-Fernández R., del Casar J. M., García C., Andicoechea A., Vázquez J., Pérez-Fernández R., Vizoso F. J. (2009). Expression and prognostic significance of metalloproteases and their inhibitors in luminal A and basal-like phenotypes of breast carcinoma. Hum. Pathol..

[40] García M. F., González-Reyes S., González L. O., Junquera S., Berdize N., Del Casar J. M., Medina M., Vizoso F. J. (2010). Comparative study of the expression of metalloproteases and their inhibitors in different localizations within primary tumours and in metastatic lymph nodes of breast cancer. Int. J. Exp. Pathol..

[41] Sauer G., Schneiderhan-Marra N., Kazmaier C., Hutzel K., Koretz K., Muche R., Kreienberg R., Joos T., Deissler H. (2008). Prediction of nodal involvement in breast cancer based on multiparametric protein analyses from preoperative core needle biopsies of the primary lesion. Clin. Cancer Res..

[42] Kim H. J., Park C. I., Park B. W., Lee H. D., Jung W. H. (2006). Expression of MT-1 MMP, MMP2, MMP9 and TIMP2 mRNAs in ductal carcinoma *in situ* and invasive ductal carcinoma of the breast. Yonsei Med. J..

[43] Dalotto-Moreno T., Croci D. O., Cerliani J. P., Martinez-Allo V. C., Dergan-Dylon S., Méndez-Huergo S. P., Stupirski J. C., Mazal D., Osinaga E., Toscano M. A. (2013). Targeting galectin-1 overcomes breast cancer-associated immunosuppression and prevents metastatic disease. Cancer Res..

[44] Fontana A., Filleur S., Guglielmi J., Frappart L., Bruno-Bossio G., Boissier S., Cabon F., Clézardin P. (2005). Human breast tumors override the antiangiogenic effect of stromal thrombospondin-1 *in vivo*. Int. J. Cancer.

[45] Wicki A., Christofori G. (2007). The potential role of podoplanin in tumour invasion. Br. J. Cancer.

[46] Malinowsky K., Böllner C., Hipp S., Berg D., Schmitt M., Becker K. F. (2010). UPA and PAI-1 analysis from fixed tissues–new perspectives for a known set of predictive markers. Curr. Med. Chem..

[47] Hildenbrand R., Schaaf A. (2009). The urokinase-system in tumor tissue stroma of the breast and breast cancer cell invasion. Int. J. Oncol..

[48] Jahkola T., Toivonen T., von Smitten K., Virtanen I., Wasenius V. M., Blomqvist C. (1999). Cathepsin-D, urokinase plasminogen activator and type-1 plasminogen activator inhibitor in early breast cancer: an immunohistochemical study of prognostic value and relations to tenascin-C and other factors. Br. J. Cancer.

[49] Sloan E. K., Ciocca D. R., Pouliot N., Natoli A., Restall C., Henderson M. A., Fanelli M. A., Cuello-Carrión F. D., Gago F. E., Anderson R. L. (2009). Stromal cell expression of caveolin-1 predicts outcome in breast cancer. Am. J. Pathol..

[50] Niemiec JA, Adamczyk A, Ambicka A, Mucha-Małecka A. M., Wysocki W, Ryś J (2013). Triple-negative, basal marker-expressing, and high-grade breast carcinomas are characterized by high lymphatic vessel density and the expression of podoplanin in stromal fibroblasts. Appl. Immunohistochem. Mol. Morphol.

[51] Del Casar JM, González-Reyes S, González LO, González JM, Junquera S, Bongera M, García MF, Andicoechea A, Serra C, Vizoso FJ. (2010). Expression of metalloproteases and their inhibitors in different histological types of breast cancer. J. Cancer Res. Clin. Oncol..

[52] O’Connell JT, Sugimoto H, Cooke VG, MacDonald BA, Mehta AI, LeBleu VS, Dewar R, Rocha RM, Brentani RR, Resnick MB (2011). VEGF-A and Tenascin-C produced by S100A4+ stromal cells are important for metastatic colonization. Proc. Natl. Acad. Sci. U. S. A..

